# Associations of the Triglyceride and Glucose Index With Hypertension Stages, Phenotypes, and Their Progressions Among Middle-Aged and Older Chinese

**DOI:** 10.3389/ijph.2023.1605648

**Published:** 2023-03-20

**Authors:** Shiyi Shan, Shuting Li, Keyao Lu, Jin Cao, Weidi Sun, Jiali Zhou, Ziyang Ren, Siyu Zhu, Leying Hou, Dingwan Chen, Peige Song

**Affiliations:** ^1^ School of Public Health and Women’s Hospital, Zhejiang University School of Medicine, Hangzhou, China; ^2^ Key Laboratory of Reproductive Health, National Health Commission of the People’s Republic of China, Institute of Reproductive and Child Health, Peking University, Beijing, China; ^3^ Department of Epidemiology and Biostatistics, School of Public Health, Peking University, Beijing, China; ^4^ School of Public Health, Hangzhou Medical College, Hangzhou, China

**Keywords:** hypertension, Chinese, triglyceride and glucose, stages, phenotypes

## Abstract

**Objectives:** To assess the associations of the triglyceride and glucose (TyG) index with hypertension stages, phenotypes, and their progressions.

**Methods:** The data originated from the China Health and Retirement Longitudinal Study. Multinomial logistic regression investigated the associations of the TyG index with hypertension stages (stage 1, stage 2), phenotypes (isolated systolic hypertension [ISH], isolated diastolic hypertension [IDH], systolic diastolic hypertension [SDH]), their progressions.

**Results:** Compared with the lowest quartile of TyG index, the highest quartile was associated with increased risks of stage 1 hypertension (OR 1.71, 95% CI 1.38–2.13), stage 2 (1.74, 1.27–2.38), ISH (1.66, 1.31–2.11), IDH (2.52, 1.26–5.05), and SDH (1.65, 1.23–2.23). Similar results were found when TyG index was a continuous variable. From 2011 to 2015, a higher baseline TyG index was associated with normotension to stage 1 (per-unit: 1.39, 1.16–1.65), normotension to ISH (per-unit: 1.28, 1.04–1.56), and normotension to IDH (per-unit: 1.94, 1.27–2.97).

**Conclusion:** The TyG index was associated with different hypertension stages, phenotypes, their progressions, and could be served as a surrogate indicator for early hypertension management.

## Introduction

Hypertension can lead to myocardial infarction, renal failure, and even death if not treated appropriately (1, 2). According to the World Health Organization report, hypertensive heart disease is one of the top 10 causes of death in upper-middle-income countries in 2019 (3). It is estimated that 1.28 billion adults aged 30–79 years are affected by hypertension in 2021 (4). In China, hypertension has evolved into a serious public health concern, as its burden grows in tandem with increased urbanization, population ageing, and exposure to unhealthy lifestyles (5–10). A review from Yin et al. reported that the prevalence of hypertension ranged from 18.0% to 44.7% in China (11). According to the *Chinese Guidelines for Prevention and Treatment of Hypertension (2018 Edition)*, hypertension statuses can be divided into hypertension and normotension, hypertension stages are classified as stage 1, stage 2, and stage 3 and hypertension phenotypes are classified as isolated systolic hypertension (ISH), isolated diastolic hypertension (IDH), and systolic diastolic hypertension (SDH) (12). Besides, as the course of hypertension progresses, changes in hypertension statues or stages or phenotypes will occur, namely, hypertension progressions. Investigating the progressions of hypertension can help to conduct dynamic management and treatment of hypertension more accurately.

The triglyceride and glucose (TyG) index has attracted increasing interest due to its excellent sensitivity (79%) and specificity (86%) for diagnosing insulin resistance (IR) (13–15). It has a high diagnostic concordance with hyperinsulinemic-euglycemic clamp (HIEC, the gold standard method for IR) and the homeostasis model assessment for insulin resistance (HOMA-IR, the most widely used surrogate indicator for IR) (14). Moreover, as a non-insulin-based index, the TyG index has an advantage of using routine laboratory measurements such as fasting triglycerides rather than insulin levels, which are less costly (16). Previous studies mainly focused on the association between the TyG index and the risk of hypertension in adults, and suggested that the TyG index was a superior indicator for hypertension than independent lipid or glycemic markers (17–20). To the best of our knowledge, limited studies have explored the associations of the TyG index with various hypertension stages and phenotypes, let alone their progressions.

To fill the research gap, this study aims to evaluate the associations of the TyG index with hypertension stages and phenotypes. And we also investigate whether the TyG index is associated with the progressions of hypertension stages and phenotypes.

## Methods

### Study Population

This study used data from the China Health and Retirement Longitudinal Study (CHARLS), a nationwide longitudinal survey of people aged 45 years or above (21). A stratified four-stage probability sampling strategy was adopted to recruit participants from 150 counties and urban districts across 28 provinces in China. The baseline survey was conducted in 2011, with the respondent rate of 80.5% for questionnaires, 78.9% for anthropometric measurements, and 67% for blood sample collection (22). Respondents were then followed up with a face-to-face computer-assisted personal interview every 2 years (2013, 2015, and 2018, respectively). Detailed study procedures can be found elsewhere (21). CHARLS was approved by the Behavioral and Social Research division of the National Institute on Aging of the National Institute of Health, the Natural Science Foundation of China, the World Bank, and Peking University. All participants provided their written informed consent to participate in this study.

**FIGURE 1:**
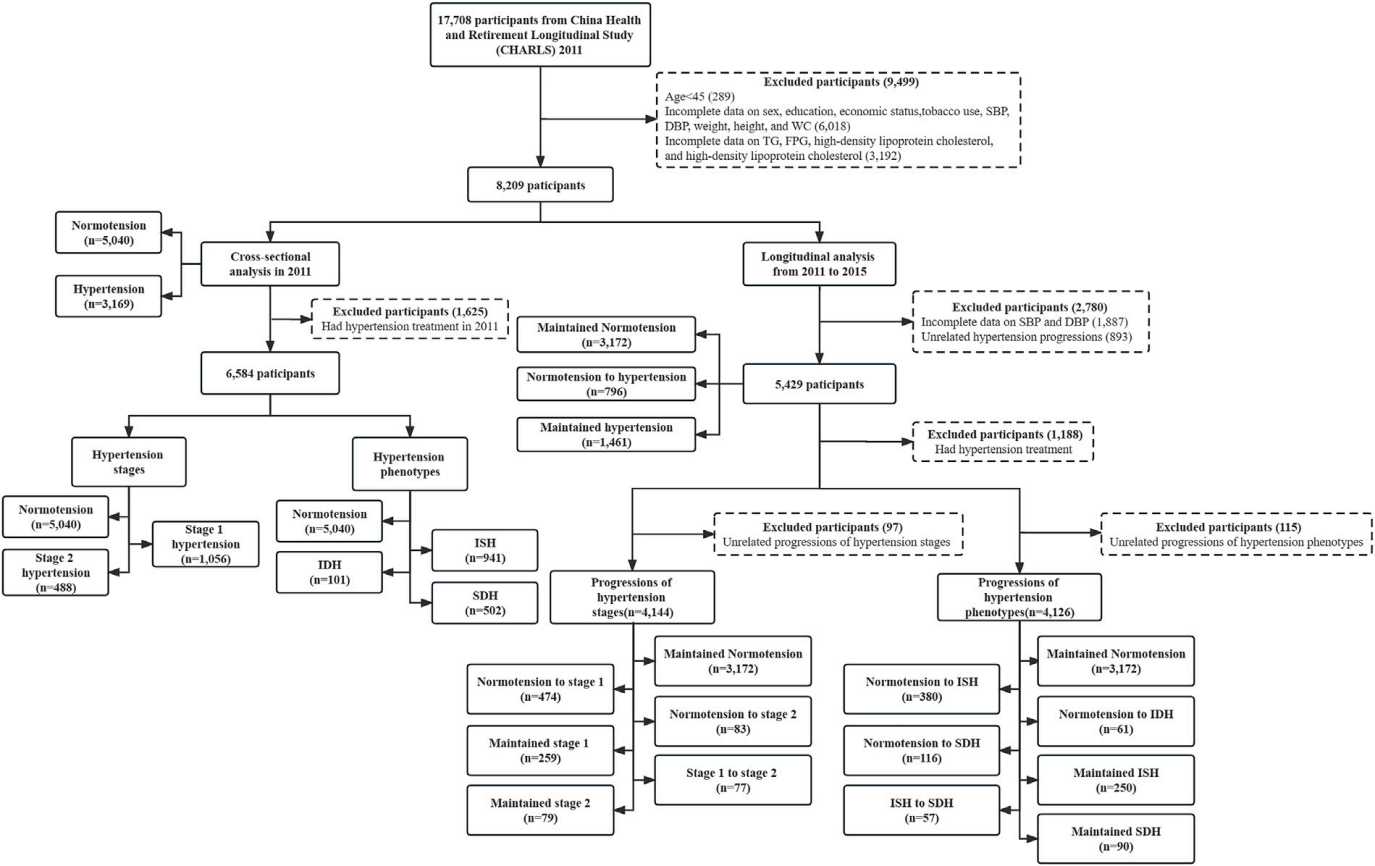
Flow chart of study participants (China Health and Retirement Longitudinal Study, China, 2011, 2015). Notes: SBP, systolic blood pressure; DBP, diastolic blood pressure; WC, waist circumstance; TG, fasting triglycerides; FPG, fasting plasma glucose; LDL-C, low-density lipoprotein cholesterol; HDL-C, high-density lipoprotein cholesterol; ISH, isolated systolic hypertension; IDH, isolated diastolic hypertension; SDH, systolic diastolic hypertension. Unrelated hypertension progressions: hypertension to normotension. Unrelated progressions of hypertension stages: stage 2 to stage 1, stage 2 to normotension, stage 1 to normotension. Unrelated progressions of hypertension phenotypes: ISH to normotension, ISH to IDH, IDH to normotension, IDH to ISH, SDH to normotension, SDH to ISH, SDH to IDH.

A total of 17,708 participants were recruited at baseline in CHARLS 2011. After excluding those under the age of 45 (*n* = 289) or with incomplete data on sex, education, economic status, tobacco use, systolic blood pressure (SBP), diastolic blood pressure (DBP), weight, height, and waist circumstance (WC) (*n* = 6,018), who had incomplete data on fasting triglycerides (TG), fasting plasma glucose (FPG), high-density lipoprotein cholesterol (HDL-C), and low-density lipoprotein cholesterol (LDL-C) (*n* = 3,192), finally 8,209 participants were included in analyses (Figure 1).

### Measurement of Blood-Based Biomarkers

Blood sample collection was done once every two follow-up periods (2011 and 2015). Biomarkers were measured using overnight fasting blood samples, which were promptly transported to the local laboratory and stored at 4°C. The blood samples were centrifugated and stored at −20°C before being transported to the China Center for Disease Prevention and Control (CDC) in Beijing and frozen at −70°C before analysis (21). Routine blood tests were performed at township hospitals or the local CDC. FPG, TG, HDL-C, and LDL-C were measured using the enzymatic colorimetric method. High sensitivity C-reactive protein (hs-CRP) was measured using the immunoturbidimetric assay.

The TyG index was calculated as $\ln\left[TG\;\left(mg/dl\right)\times FPG\;\left(mg/dl\right)/2\right]$ (13).

### Definition of Hypertension Stages, Phenotypes and Progressions

Blood pressure (BP) was measured three times in a sitting position at a 45-s interval using an electronic monitor (Omron model HEM-7200). The average of the three measurements was calculated to the nearest 0.1 mmHg for analysis. Hypertension statuses were divided into hypertension and normotension. Hypertension was defined as mean SBP ≥ 140 mmHg and/or mean DBP ≥ 90 mmHg and/or having hypertension treatments (12, 21). Normotension was defined as SBP < 140 mmHg and DBP < 90 mmHg. After excluding those with antihypertensive medications, hypertension stages were further classified as stage 1 hypertension (SBP ≥ 140 mmHg and < 159 mmHg and/or DBP ≥ 90 mmHg and < 99 mmHg), stage 2 hypertension (SBP ≥ 160 mmHg and < 179 mmHg and/or DBP ≥ 100 mmHg and < 109 mmHg), and stage 3 hypertension (SBP ≥ 180 mmHg and/or DBP ≥ 110 mmHg). Given the sample size constraints, we combined stage 2 and stage 3 hypertension into stage 2 hypertension. Hypertension phenotypes were also classified as ISH (SBP ≥ 140 mmHg and DBP < 90 mmHg), IDH (SBP < 140 mmHg and DBP ≥ 90 mmHg), and SDH (SBP ≥ 140 mmHg and DBP ≥ 90 mmHg). More details are shown in Supplementary Table S1. The progressions of hypertension statuses were classified as maintained normotension, normotension to hypertension, and maintained hypertension. The progressions of hypertension stages were classified as maintained normotension, normotension to stage 1, normotension to stage 2, maintained stage 1, stage 1 to stage 2, and maintained stage 2. The progressions of hypertension phenotypes were classified as maintained normotension, normotension to ISH, normotension to IDH, normotension to SDH, maintained ISH, ISH to SDH, and maintained SDH.

### Definition of Covariates

Anthropometric measurements, including height, weight, and WC, were made at every 2-year follow-up. Information on age, sex, residence, education, economic status, tobacco use, and alcohol consumption were collected through questionnaires. The residence was classified as urban and rural according to the location of the participants. Education was divided into illiterate, primary school and below, and junior high school or above. Economic status was evaluated by the natural logarithm of *per capita* expenditures (ln [PCE]) (23). The bottom, middle, and top tertile of ln (PCE) indicated poor, middle, and rich economic status, respectively. Tobacco use was categorized as never smoking and ever smoking (including former smokers and current smokers). Likewise, alcohol consumption was classified as never drinking and ever drinking (including former alcohol consumers and current alcohol consumers). Body mass index (BMI) was calculated as $\left(weight\;\left[kg\right]\;/{\left[height\;[m\right]}^{2}\right)$ . The categories of general obesity included normal (BMI < 24 kg/m^2^), overweight (BMI ≥ 24 kg/m^2^ and BMI < 28 kg/m^2^), and obesity (BMI ≥ 28 kg/m^2^) (24). Diabetes was defined as FPG ≥ 7.0 mmol/L, and/or random plasma glucose ≥ 11.1 mmol/L, and/or HbA1c ≥ 6.5%, and/or self-reported treatment (25).

### Statistical Analysis

The normality test showed that all continuous variables were skewed. Characteristics of participants at baseline (CHARLS 2011) were described in the form of the median with interquartile range (IQR) for continuous variables with skewed distributions, and number with percent (%) for categorical variables. The basic characteristics of participants between the included participants and excluded participants, between normotension and hypertension, among different hypertension stages, hypertension phenotypes, and hypertension progressions were compared using Wilcoxon rank-sum test for continuous variables and the Chi-square test for categorical variables.

The included participants were divided into four groups based on their TyG index levels (quartile 1 [Q1], quartile 2 [Q2], quartile 3 [Q3] and quartile 4 [Q4]) (18, 26). In the data analysis, the TyG index was considered as a continuous (per-unit) variable and converted to a categorical (quartiles) variable.

For the cross-sectional analysis in CHARLS 2011, we used binomial logistic regression to assess the associations between TyG index and hypertension statuses. Meanwhile, we used multinomial logistic regressions to investigate the associations of the TyG index with hypertension stages and phenotypes, with normotension as reference. The model was adjusted for age, sex, residence, education, economic status, tobacco use, alcohol consumption, general obesity, WC, LDL-C, HDL-C, and hs-CRP. Meanwhile, considering the sex-TyG index interaction, we conducted sex-stratified multinomial logistic regression as well (27). The goodness of fit of the multinomial logistic regression model has been verified using the Hosmer-Lemeshow Goodness-of-Fit Test. Consider the impacts of diabetes, hypoglycemic agents, insulin, or dyslipidemia drugs on glucose, lipid metabolism, and BP. Sensitivity analyses were also conducted as follows to explore the stability of our findings: 1) excluding people with diabetes; 2) further adjusted diabetes and dyslipidemia treatment. We further investigated the optimal cutoff point of TyG index for prediction of hypertension according to best Youden index (YI, sensitivity+specificity−1). The receiver operating characteristics (ROC) curve and the area under the curve (AUC) of TyG index were also conducted.

For the longitudinal analysis using CHARLS 2011 and 2015, we excluded those who had hypertension treatments to ensure proper classification of hypertension stages and phenotypes. Participants interviewed in both waves with complete data on SBP and DBP were included to further investigate the associations between the TyG index and hypertension progressions. Those who had non-attenuated hypertension progression patterns including maintained normotension, normotension to hypertension, and maintained hypertension based on their hypertension statuses from 2011 to 2015 were included. Subsequently, we selected participants who had non-attenuated hypertension stages (i.e., maintained normotension, normotension to stage 1, normotension to stage 2, maintained stage 1, stage 1 to stage 2, and maintained stage 2) and phenotypes (i.e., maintained normotension, normotension to ISH, normotension to IDH, normotension to SDH, maintained ISH, ISH to SDH, and maintained SDH) to explore whether the TyG index was associated with hypertension stages and phenotypes progressions, respectively. Those who had unrelated progressions, which referred to attenuated hypertension progression patterns (i.e., hypertension to normotension; stage 2 to stage 1, stage 2 to normotension, stage 1 to normotension; ISH to normotension, ISH to IDH, IDH to normotension, IDH to ISH, SDH to normotension, SDH to ISH, SDH to IDH), were excluded, accordingly. The model and adjustments were consistent with those above. All of these analyses were conducted using sex-stratified multinomial logistic regression with maintained normotension as reference.

This study was done following the Strengthening the Reporting of Observational Studies in Epidemiology (STROBE) guidelines (28). SAS statistical software (version 9.4; SAS Institute Inc., Cary, NC, United States) was used. All analyses were two-sided, and statistical significance was defined as a *p*-value <0.05.

## Results

### Characteristics of Baseline Study Population

Characteristic comparisons between the included (*n* = 8,209) and excluded (*n* = 9,499) participants were shown in Supplementary Table S2. The average age was 59.00 (52.00–65.00) of the included participants, with a greater proportion of female (53.0%). More than half of the participants lived in rural area (65.7%), never smoked (60.4%), never had alcohol consumption (74.7%). Table 1 showed that of the 8,209 individuals included in CHARLS 2011, 5,040 (61.4%) had normotension, and 3,169 (38.6%) had hypertension. Among them, 406 participants received diabetes treatment (took medicine or insulin injection) and dyslipidemia treatment (took medicine or other treatment). The median TyG index was 8.52 (IQR: 8.17–8.94) for normotension, and 8.74 (IQR: 8.34–9.20) for hypertension, with *p* values <0.05. Significant differences in age, residence, education, economic status, alcohol consumption, general obesity, WC, LDL-C, HDL-C, and hs-CRP between different hypertension statuses were also observed (all *p* values <0.05).

**TABLE 1:** Characteristics of study participants by hypertension statuses in 2011 (*N* = 8,209) (China Health and Retirement Longitudinal Study, China, 2011).

Characteristics	Normotension	Hypertension	*p*-value
No. of Participants	5,040 (61.4)	3,169 (38.6)	
Age, year	57.00 (50.00–63.00)	61.00 (55.00–69.00)	<0.001
Sex			0.068
Male	2,410 (47.8)	1,450 (45.8)	
Female	2,630 (52.2)	1,719 (54.2)	
Residence			<0.001
Urban	1,634 (32.4)	1,182 (37.3)	
Rural	3,406 (67.6)	1,987 (62.7)	
Education			<0.001
Illiterate	1,326 (26.3)	1,019 (32.2)	
Primary school and below	2,092 (41.5)	1,318 (41.6)	
Junior high school or above	1,622 (32.2)	832 (26.3)	
Economic status			0.019
Poor	1,650 (32.7)	1,091 (34.4)	
Middle	1,696 (33.7)	1,107 (34.9)	
Rich	1,694 (33.6)	971 (30.6)	
Tobacco use			0.095
Never smoking	3,008 (59.7)	1,950 (61.5)	
Ever smoking	2,032 (40.3)	1,219 (38.5)	
Alcohol consumption			0.032
Never drinking	3,723 (73.9)	2,408 (76.0)	
Ever drinking	1,317 (26.1)	761 (24.0)	
General obesity			<0.001
Normal	3,308 (65.6)	1,508 (47.6)	
Overweight	1,350 (26.8)	1,089 (34.4)	
Obesity	382 (7.6)	572 (18.1)	
Diabetes treatment or dyslipidemia treatment	157 (61.3)	249 (38.7)	<0.001
WC, cm	82.80 (76.40–89.40)	88.20 (81.00–95.80)	<0.001
LDL-C, mg/dL	113.27 (92.78–134.92)	116.37 (94.72–141.50)	<0.001
HDL-C, mg/dL	50.64 (41.37–60.70)	47.55 (39.05–57.99)	<0.001
Hs-CRP, mg/L	0.90 (0.50–1.90)	1.29 (0.66–2.69)	<0.001
The TyG index	8.52 (8.17–8.94)	8.74 (8.34–9.20)	<0.001

Notes: WC, waist circumstance; LDL-C, low-density lipoprotein cholesterol; HDL-C, high-density lipoprotein cholesterol; Hs-CRP, high sensitivity C-reactive protein; The TyG index, triglyceride-glucose index. Values are presented as number (N) with percent (%) for categorical variables or median with interquartile range (IQR) for continuous variables.

### Cross-Sectional Analyses

For the cross-sectional analysis in CHARLS 2011, we found that when compared with the bottom quartile of baseline TyG index, a higher quartile of TyG index was significantly associated with hypertension (adjusted odds ratio [aOR] 1.17, 95% confidence interval [CI] 1.01–1.35 for Q2; aOR 1.48, 95% CI 1.28–1.72 for Q3; aOR 1.90, 95% CI 1.63–2.23 for Q4). Similar results were also found when the TyG index was used as a continuous variable and in the sex-stratified analyses (Table 2). In Supplementary Tables S3, S4, the sensitivity analyses of the TyG index with hypertension in 2011 among population without diabetes and further adjusted for diabetes and dyslipidemia treatment showed similar results in line with our primary analysis. Supplementary Figure S1 presented the ROC curve of the TyG index for prediction of hypertension, with AUC of 0.595. According to YI, the optimal cutoff point of TyG index in predicting hypertension was 8.637.

**TABLE 2:** Association between triglyceride-glucose index and hypertension in 2011 (China Health and Retirement Longitudinal Study, China, 2011).

Variables	Overall (N = 8,209)		Male (N = 3,860)		Female (N = 4,349)	
	Crude OR (95% CI)	Adjusted OR (95% CI)	Crude OR (95% CI)	Adjusted OR (95% CI)	Crude OR (95% CI)	Adjusted OR (95% CI)
Quartile 1	Reference	Reference	Reference	Reference	Reference	Reference
Quartile 2	1.26 (1.11–1.44)	1.17 (1.01–1.35)	1.13 (0.94–1.37)	1.07 (0.88–1.32)	1.38 (1.15–1.66)	1.17 (0.96–1.42)
Quartile 3	1.76 (1.55–2.01)	1.48 (1.28–1.72)	1.48 (1.23–1.79)	1.33 (1.08–1.64)	2.03 (1.70–2.43)	1.57 (1.28–1.93)
Quartile 4	2.39 (2.10–2.72)	1.90 (1.63–2.23)	1.98 (1.64–2.38)	1.64 (1.31–2.05)	2.73 (2.29–3.27)	1.99 (1.59–2.48)
TyG-continuous	1.62 (1.52–1.74)	1.45 (1.33–1.58)	1.50 (1.36–1.65)	1.38 (1.23–1.56)	1.75 (1.59–1.93)	1.48 (1.31–1.67)

Notes: TyG, triglyceride-glucose; OR, odds ratio; CI, confidence interval; OR values were adjusted for age, sex, residence, education, economic status, tobacco use, alcohol consumption, body mass index, waist circumstance, low-density lipoprotein cholesterol, high-density lipoprotein cholesterol, and high sensitivity C-reactive protein.

After excluding participants who had hypertension treatment in 2011 (*n* = 1,625), a total of 6,584 participants were finally included in the classification of hypertension stages or phenotypes, according to SBP and DBP levels. Baseline characteristics of the remaining 6,584 participants by hypertension stages and phenotypes were shown in Supplementary Tables S5, S6, respectively. We also found that compared with people with the lowest quartile of baseline exposure, those with a higher TyG index had an increased risk of stage 1 hypertension (aOR 1.26, 95% CI 1.02–1.55 for Q3; aOR 1.71, 95% CI 1.38–2.13 for Q4) and stage 2 hypertension (aOR 1.52, 95% CI 1.13–2.04 for Q3; aOR 1.74, 95% CI 1.27–2.38 for Q4). Furthermore, the TyG index was also positively associated with ISH (aOR 1.35, 95% CI 1.08–1.68 for Q3; aOR 1.66, 95% CI 1.31–2.11 for Q4), IDH (aOR 2.04, 95% CI 1.03–4.04 for Q3; aOR 2.52, 95% CI 1.26–5.05 for Q4), and SDH (aOR 1.65, 95% CI 1.23–2.23 for Q4) (Table 3). Similarly, a higher TyG index (per-unit) was significantly associated with the development of stage 1 hypertension (aOR 1.43, 95% CI 1.27–1.61), stage 2 hypertension (aOR 1.42, 95% CI 1.20–1.67), ISH (aOR 1.41, 95% CI 1.24–1.61), IDH (aOR 1.78, 95% CI 1.30–2.45), SDH (aOR 1.35, 95% CI 1.15–1.58). However, when stratified by sex, neither as a continuous variable nor as a categorical variable of the TyG index had a significant association with stage 2 hypertension in males.

**TABLE 3:** Association of the triglyceride-glucose index with hypertension stages and hypertension phenotypes in 2011 (China Health and Retirement Longitudinal Study, China, 2011).

	Stage 1 hypertension		Stage 2 hypertension		ISH		IDH		SDH	
	Crude OR (95% CI)	Adjusted OR (95% CI)	Crude OR (95% CI)	Adjusted OR (95% CI)	Crude OR (95% CI)	Adjusted OR (95% CI)	Crude OR (95% CI)	Adjusted OR (95% CI)	Crude OR (95% CI)	Adjusted OR (95% CI)
Overall (N = 6,584)										
Quartile 1	Reference	Reference	Reference	Reference	Reference	Reference	Reference	Reference	Reference	Reference
Quartile 2	1.09 (0.89–1.33)	1.10 (0.89–1.35)	1.12 (0.84–1.50)	1.14 (0.85–1.54)	1.07 (0.87–1.32)	1.10 (0.88–1.37)	1.60 (0.82–3.14)	1.50 (0.75–3.00)	1.09 (0.82–1.44)	1.07 (0.80–1.42)
Quartile 3	1.28 (1.06–1.56)	1.26 (1.02–1.55)	1.56 (1.19–2.05)	1.52 (1.13–2.04)	1.37 (1.13–1.68)	1.35 (1.08–1.68)	2.14 (1.12–4.09)	2.04 (1.03–4.04)	1.25 (0.95–1.64)	1.20 (0.90–1.62)
Quartile 4	1.67 (1.38–2.01)	1.71 (1.38–2.13)	1.72 (1.32–2.25)	1.74 (1.27–2.38)	1.50 (1.23–1.83)	1.66 (1.31–2.11)	2.99 (1.61–5.56)	2.52 (1.26–5.05)	1.86 (1.44–2.40)	1.65 (1.23–2.23)
TyG-continuous	1.37 (1.24–1.51)	1.43 (1.27–1.61)	1.38 (1.20–1.58)	1.42 (1.20–1.67)	1.28 (1.15–1.42)	1.41 (1.24–1.61)	1.87 (1.44–2.43)	1.78 (1.30–2.45)	1.46 (1.28–1.67)	1.35 (1.15–1.58)
Male (N = 3,154)										
Quartile 1	Reference	Reference	Reference	Reference	Reference	Reference	Reference	Reference	Reference	Reference
Quartile 2	1.07 (0.81–1.42)	1.08 (0.81–1.45)	1.01 (0.68–1.50)	1.05 (0.70–1.58)	1.00 (0.74–1.35)	1.06 (0.78–1.46)	1.23 (0.51–3.00)	1.02 (0.41–2.55)	1.12 (0.76–1.65)	1.11 (0.74–1.64)
Quartile 3	1.15 (0.87–1.52)	1.15 (0.85–1.55)	1.15 (0.78–1.69)	1.24 (0.82–1.88)	0.99 (0.73–1.34)	1.11 (0.80–1.54)	1.49 (0.63–3.51)	1.16 (0.47–2.87)	1.39 (0.96–2.01)	1.34 (0.91–1.99)
Quartile 4	1.68 (1.29–2.19)	1.74 (1.28–2.36)	1.22 (0.82–1.79)	1.33 (0.85–2.09)	1.26 (0.95–1.69)	1.65 (1.17–2.32)	2.84 (1.30–6.18)	1.66 (0.68–4.04)	1.81 (1.27–2.59)	1.62 (1.07–2.44)
TyG-continuous	1.35 (1.17–1.54)	1.42 (1.20–1.67)	1.15 (0.94–1.41)	1.25 (0.98–1.60)	1.14 (0.97–1.33)	1.36 (1.12–1.65)	2.00 (1.44–2.77)	1.65 (1.08–2.50)	1.39 (1.16–1.65)	1.31 (1.06–1.62)
Female (N = 3,430)										
Quartile 1	Reference	Reference	Reference	Reference	Reference	Reference	Reference	Reference	Reference	Reference
Quartile 2	1.24 (0.94–1.64)	1.17 (0.87–1.57)	1.34 (0.89–2.04)	1.21 (0.79–1.87)	1.32 (1.00–1.76)	1.22 (0.90–1.67)	3.13 (1.01–9.75)	3.21 (1.01–10.20)	1.00 (0.65–1.53)	0.91 (0.58–1.41)
Quartile 3	1.46 (1.11–1.92)	1.33 (0.98–1.79)	2.14 (1.45–3.16)	1.74 (1.14–2.65)	1.72 (1.31–2.27)	1.47 (1.08–2.01)	4.16 (1.37–12.59)	4.40 (1.38–14.05)	1.28 (0.85–1.03)	1.13 (0.73–1.76)
Quartile 4	1.81 (1.39–2.37)	1.79 (1.30–2.46)	2.46 (1.67–3.60)	2.03 (1.30–3.19)	1.84 (1.40–2.43)	1.81 (1.29–2.54)	4.09 (1.34–12.49)	3.97 (1.17–13.43)	2.14 (1.47–3.12)	1.74 (1.11–2.72)
TyG-continuous	1.40 (1.22–1.62)	1.44 (1.21–1.71)	1.61 (1.34–1.94)	1.45 (1.15–1.84)	1.39 (1.21–1.61)	1.43 (1.19–1.72)	1.73 (1.13–2.63)	1.73 (1.04–2.87)	1.61 (1.32–1.97)	1.40 (1.10–1.78)

Notes: TyG, triglyceride-glucose; ISH, isolated systolic hypertension; IDH, isolated diastolic hypertension; SDH, systolic-diastolic hypertension; OR, odds ratio; CI, confidence interval; OR values were adjusted for age, sex, residence, education, economic status, tobacco use, alcohol consumption, general obesity, waist circumstance, low-density lipoprotein cholesterol, high-density lipoprotein cholesterol, and high sensitivity C-reactive protein.

### Longitudinal Analyses

In the longitudinal study using CHARLS 2011 and 2015, participants with incomplete data on SBP and DBP (*n* = 1,887) or unrelated hypertension progressions (*n* = 893) were further excluded, leaving 5,429 participants for longitudinal analyses. During the 4-year follow-up, 796 individuals (14.7%) developed hypertension (Supplementary Table S7). Participants who were in the highest quartile TyG index were more likely to have the progression of normotension to hypertension (normotension to hypertension vs. maintained normotension: aOR 1.48, 95% CI 1.14–1.92) and maintained hypertension (maintained hypertension vs. maintained normotension: aOR 2.16, 95% CI 1.74–2.69), compared with those in the lowest quartile TyG index, as shown in Supplementary Table S8. Associations of the per-unit TyG index with the progression of normotension to hypertension (aOR 1.34, 95% CI 1.16–1.54) and maintained hypertension (aOR 1.59, 95% CI 1.41–1.79) were also found when compared with maintained normotension. However, when stratified by sex, there was no evidence of significant associations between the TyG index (per-unit) and normotension to hypertension (aOR 1.18, 95% CI 0.96–1.45), compared with maintained normotension in females (Supplementary Table S8).

**FIGURE 2:**
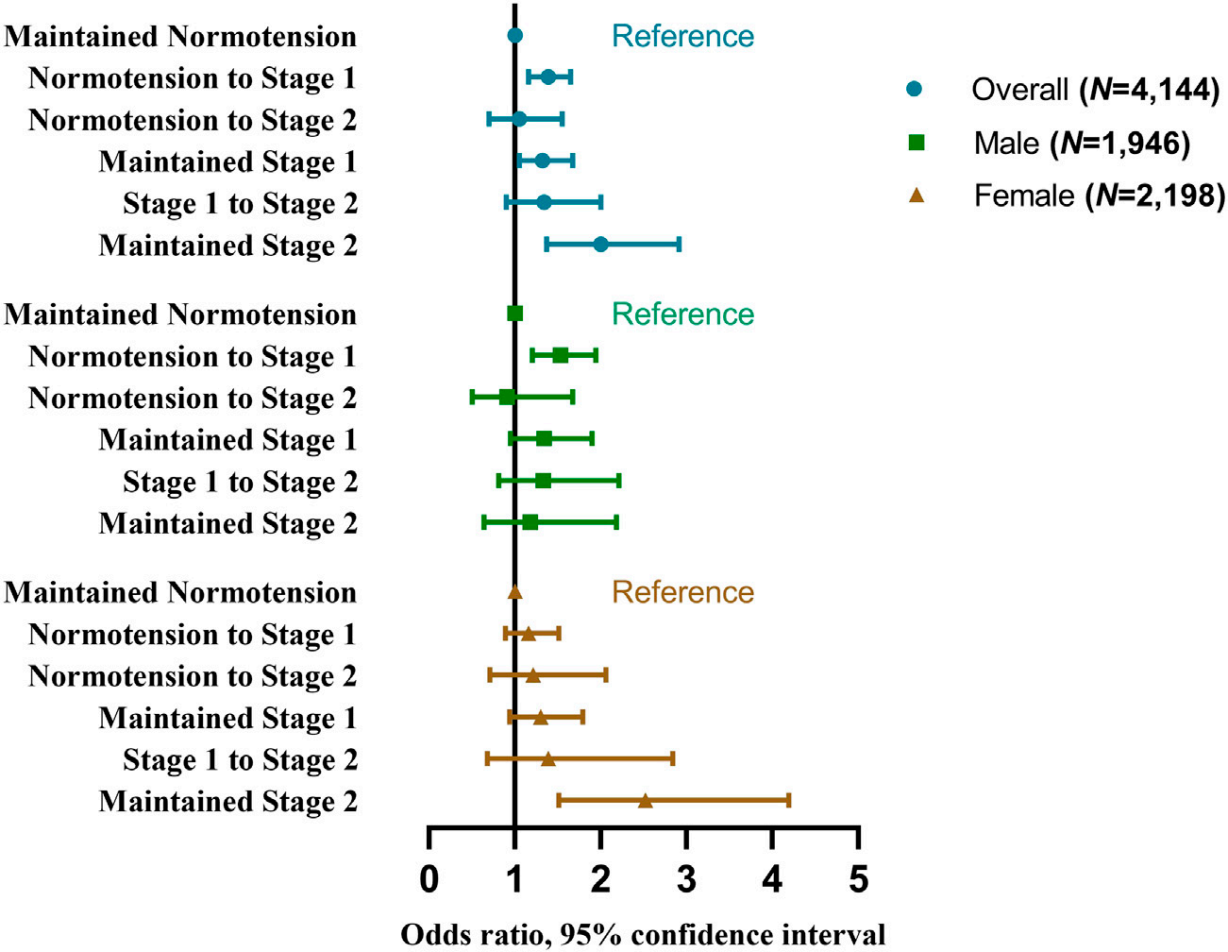
Odds ratios and 95% confidence interval of the progressions of hypertension stages by the triglyceride-glucose index (China Health and Retirement Longitudinal Study, China, 2011–2015).

Furthermore, after excluding participants who had hypertension treatment in 2011 and 2015 (*n* = 1,188), with unrelated progressions of hypertension stages (*n* = 97) and unrelated progressions of hypertension phenotypes (*n* = 115), 4,144 and 4,126 participants were enrolled separately to explore whether the TyG index was associated with the progressions of hypertension stages and phenotypes. Characteristics of these participants were described in Supplementary Tables S9, S10, respectively. As shown in Supplementary Table S11 and Figure 2, individuals with a higher baseline TyG index were more likely to have progressions of normotension to stage 1 (for highest quartile vs. lowest quartile: aOR 1.45, 95%CI 1.05–2.00; for per-unit: aOR 1.39, 95% CI 1.16–1.65), maintained stage 1 (for highest quartile vs. lowest quartile: aOR 1.68, 95%CI 1.10–2.56; for per-unit: aOR 1.32, 95% CI 1.05–1.67), and maintained stage 2 (for highest quartile vs. lowest quartile: aOR 2.62, 95%CI 1.23–5.62; for per-unit: aOR 2.00, 95% CI 1.37–2.91).

**FIGURE 3:**
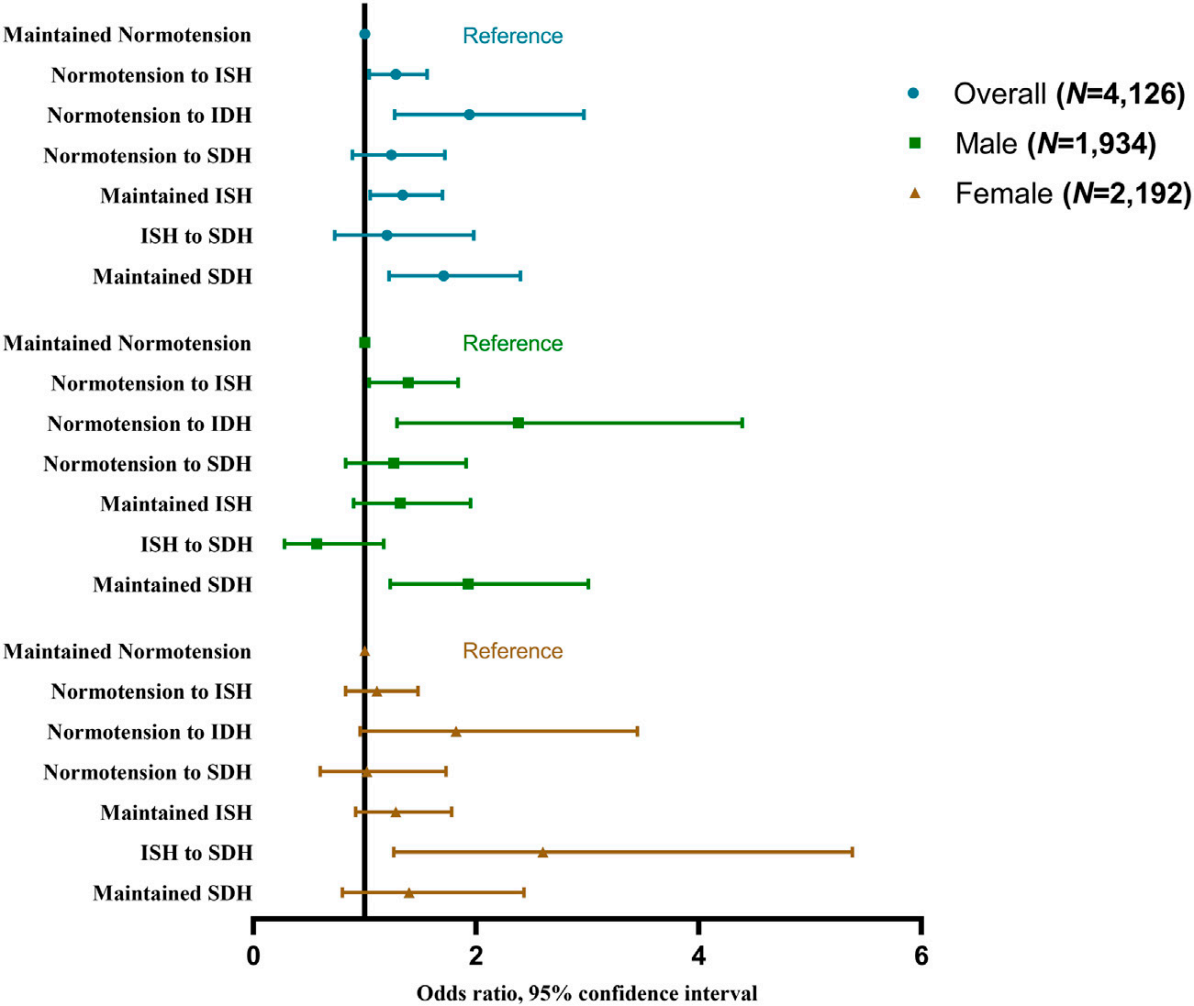
Odds ratios and 95% confidence interval of the progressions of hypertension phenotypes by the triglyceride-glucose index (China Health and Retirement Longitudinal Study, China, 2011–2015). Notes: ISH, isolated systolic hypertension; IDH, isolated diastolic hypertension; SDH, systolic diastolic hypertension.

In addition, we also found significant associations of the TyG index with normotension to ISH (per-unit: aOR 1.28, 95% CI 1.04–1.56), normotension to IDH (for highest quartile vs. lowest quartile: aOR 3.46, 95%CI 1.42–8.44; for per-unit: aOR 1.94, 95% CI 1.27–2.97), maintained ISH (for highest quartile vs. lowest quartile: aOR 1.63, 95%CI 1.03–2.57; for per-unit: aOR 1.34, 95% CI = 1.05–1.70), and maintained SDH (for highest quartile vs. lowest quartile: aOR 2.66, 95%CI 1.36–5.20; for per-unit: aOR 1.71, 95% CI 1.22–2.40) as shown in Supplementary Table S12 and Figure 3.

## Discussion

In this study, we found significant associations between a higher TyG index with increased risks of every hypertension stage and phenotype. Increased TyG index was also associated with the progressions of hypertension status (including normotension to hypertension and maintained hypertension), stages (including normotension to stage 1, maintained stage 1 and maintained stage 2) and phenotypes (including normotension to ISH, normotension to IDH, maintained ISH and maintained SDH).

Our results are consistent with previous findings, which discovered that a higher TyG index is associated with higher odds of hypertension (18, 19, 26, 29, 30). Because of the TyG index’s high sensitivity and specificity, it has been proposed as a prominent indicator of IR (14, 15). And the association between IR and hypertension has been verified in some researches (31–33). So, several potential mechanisms *via* IR may explain our main results. IR may cause hyperinsulinemia and elevate BP by stimulating the sympathetic nervous system and the adrenergic system (34–36). Increased insulin enhances insulin-mediated glucose metabolism in neurons and stimulates the sympathetic centre in the brain stem, resulting in higher BP (37). What’s more, sympathetic excitement can generate increased catecholamine levels, which thicken vascular smooth muscle and lead to hypertension (38). IR may also promote the release of angiotensin II by activating the renin-angiotensin system (RAS). Angiotensin II can inhibit the phosphatidylinositol 3-kinase pathway while activating the mitogen-activated protein kinase pathway and finally contribute to hypertension (36, 39, 40). Meanwhile, elevated angiotensin II promotes adrenal aldosterone secretion and leads to sodium retention, plasma volume expansion, and non-genetic mineralocorticoid receptor mediation, all of which play an important role in hypertension pathogenesis (41, 42).

Our study supplemented previous findings and revealed that the TyG index was associated with multiple hypertension stages and phenotypes, as well as their progressions. Kaze et al. (43) found a positive association between the development of IR and BP progressions, which aligns with our findings. In particular, we found that a higher TyG index was associated with minor hypertension progressions (i.e., normotension to stage 1, normotension to ISH, and normotension to IDH), implying that the TyG index could be a potential early screening indicator for hypertension. Given our sample limitations, we conducted our study in the general population but not in patients with hypertension, which may pull down the average TyG level and lead to the non-significant association of the TyG index with normotension to stage 2 and normotension to SDH. Therefore, the association of the TyG index with hypertension progressions across a broader range warrants further validation in larger-scale studies. However, in the sex-stratified analysis, we found that a higher TyG index was significantly associated with stage 2 hypertension in females, but not in males. The sex-stratified results may be related to the change in female estrogen levels. Estradiol (E2), is the most potent form of estrogen that almost always exists in the body of women of reproductive age (44). During a woman’s fertile life, the average level of total estrogen is 100–250 pg/mL, while the E2 concentration in the postmenopausal circulation drops to 10 pg/mL (45). Research has shown that endogenous estrogen may play a role in higher insulin sensitivity in females by decreasing gluconeogenesis and glycogenolysis (46, 47). Additionally, animals and humans lacking endogenous estrogen synthesis exhibit IR, which can be treated by estrogen supplement (47). Our study included females who were older than 45 years, and most of them were postmenopausal. Their dropping estrogen level resulted in a decrease of insulin sensitivity, making them susceptible to IR. Moreover, we found that a higher TyG index was significantly correlated with different progressions of hypertension in males (normotension to stage 1, normotension to ISH, normotension to IDH, maintained SDH), but not in females. Differences in the preferred location of fat storage between men and women may also affect the development of IR (48, 49). Women are more likely to store fat subcutaneously, while men store more abdominal fat with a higher amount of abdominal visceral fat and ectopic fat (50, 51). Therefore, the level of visceral fat storage will be higher in men than in women with the same BMI. High visceral fat mass is associated with an increased risk of IR and abdominal obesity causes IR by stimulating the formation of metabolites from lipids, hormones and cytokines (52), which may lead to sex differences in the progression of hypertension.

### Strengths and Limitations

The principal strength of this study is that we are the first to explore the associations of the TyG index with various hypertension stages and phenotypes, as well as their progressions, in a large Chinese general population. Since the TyG index is a recently proposed and high-profile indicator, our findings assist to accelerate the clinical application of the TyG index to facilitate early hypertension screening. Additionally, hypertension is known to be common in elderly with higher risks of other diseases, such as metabolic syndrome, type 2 diabetes, and cardiovascular diseases (26, 53). Our results indicate that the TyG index is helpful in predicting the risk of hypertension in elderly, suggesting that the TyG index may have a valuable application value in the clinic risk assessment of other diseases. Moreover, we used data from the CHARLS, which has broad geographic coverage, rigorous implementation procedures, and nationwide samples to ensure the national representation of our conclusion.

Despite these strengths, several limitations need to be recognized. First, the TyG index was only assessed in 2011, and its changes during follow-up were not taken into account. Second, there were significant characteristic differences between included and excluded participants (Supplementary Table S2), which may lead to selection bias and affect generalizability. Third, since all participants were Chinese adults aged 45 and over, our findings cannot be directly extrapolated to younger groups. Fourth, we merged stage 2 and stage 3 hypertension into stage 2 hypertension due to the limited sample size, which may result in misjudging the association between the TyG index and stage 2 hypertension, or the progressions. Moreover, the participants in our study are the general population but not the patients with hypertension, which may pull down the average TyG level and lead to the non-significant association of the TyG index with normotension to stage 2 and normotension to SDH. More high-quality epidemiologic investigations and primary prevention studies are needed to explore the role of TyG index in the progression of hypertension. Besides, the BP variability and the different environments, times of measurement (seasonal variations or diurnal variations) may lead to inaccuracy of BP values (54–57). Finally, even though the model we used was adjusted for multiple covariates, certain possible confounding factors, such as exercise habits and family history of hypertension, were not included.

### Conclusion

In conclusion, our study revealed that a higher TyG index was significantly associated with hypertension stages, phenotypes, and their progressions. Given the rising prevalence of hypertension and diabetes in China, the TyG index may serve as a useful indicator for the early prevention of hypertension and help to identify persons at higher risk of hypertension.
